# Antioxidant activity **and total phenolic content of** *Boerhavia elegans* (choisy) **grown in Baluchestan, Iran**

**Published:** 2015

**Authors:** Zahra Sadeghi, Jafar Valizadeh, Omid Azyzian Shermeh, Maryam Akaberi

**Affiliations:** 1 *Department of Production and Utilization of Medicinal Plants, Faculty of Agricultural and Natural Resources, Higher Educational Complex of Saravan, Saravan, I. R. Iran*; 2 *Department of Biology, University of Sistan & Baluchestan, Zahedan, I. R. Iran*; 3 *Biotechnology Research Center and School of Pharmacy, Mashhad University of Medical Sciences, Mashhad, I. R. Iran*

**Keywords:** *Boerrhaviaelegans*, *Antioxidants*, *DPPH*, *FRAP*, *Totalphenoliccontent*

## Abstract

**Objective:** *Boerhaavia elegans *L. (Nyctaginaceae) is a medicinal plant used for the treatment of kidney disorders, urinary tract disorders and blood purification in Baluch tribe. The aim of present study is to evaluate the antioxidant property of* B. elegans* species for the first time.

**Materials and Methods:** Different parts (leaf, stem and fruit) of the plant were extracted by using various solvents (water, methanol, chloroform and ethyl acetate) and evaluated for their antioxidant activity using DPPH (2, 2-diphenyl-1 picryl hydrazyl) and FRAP (ferric reducing antioxidant power) methods. In addition, total phenolic content was determined by Folin–Ciocalteu reagent.

**Results**: Antioxidant results were expressed as IC_50_. The antioxidant power in DPPH and FRAP assay were evaluated as shown in decreasing order: Methanolic extract > Aqueous extract > Ethyl acetate extract > Chloroform extract, for all parts of the plant. In both methods of antioxidant assay and Folin-Ciocalteu method, methanolic extract of leaf exhibited the highest activity and the most phenolic content IC_50_= 6.85 ppm and 16.41 mg GA/g d w respectively. Total phenolic content had a positive relationship with antioxidant capacity in extracts and there was a high correlation (r=1.00, p<0.01) between antioxidant activities as determined by both antioxidant assays for various parts.

**Conclusion:** The results of the experiments showed that *B. elegans *extract had significant antioxidant effects. This high antioxidant activity may be linked to phenolic contents of the plant but complementary investigations are suggested in order to determine active elements.

## Introduction

About 5% or more of the inhaled oxygen (O_2_) is converted to reactive oxygen species such as O_2_^-^, H_2_O_2_, and OH. Antioxidants can act by scavenging reactive oxygen species, inhibiting their formation, binding transition metal ions and preventing formation of OH and/or decomposition of lipid hydroperixides, which could lead to the repairing of damages (Gupta et al., 2006 ▶). 

Antioxidants play an important role in providing protection to humans against infection and degenerative diseases. Antioxidants are classified into two major categories, natural and synthetic. Several synthetic antioxidants, such as butylated hydroxyl anisole and hydroxyl toluene, are commercially available and are used in 50-200 ppm in foods and have many side effects such as mutagenesis carcinogenic in human beings (Ebrahimzadeh et al., 2008 ▶; Ghanbari et al., 2006 ▶).

 Natural antioxidants are safe and also bioactive. Among the various natural products, phenolic compounds are natural antioxidants that have the character of quenching oxygen-derived free radicals by donating a hydrogen atom or an electron to the free radical. In addition, these compounds have anti-inflammatory, anti-carcinogenic and anti-atherosclerotic activities (Sonboli et al., 2010 ▶; Huang et al., 2010 ▶). Therefore, recently, wide investigations have been done for identification of plants with antioxidant activity that may be used for treatment of various diseases in human (Jayavelu et al., 2013 ▶).

*Boerhavia* (Nyctaginaceae) is a genus with about 40 species, almost all of which are dispersed widely in tropical and subtropical areas on gravelly plains, or on rocky slopes (Fosberg, 1978 ▶; Spellenberg, 2012 ▶). Among 40 species of *Boerhavia*, 3 species are found in Iran, namely *B. repens,B. diffusa* and *B. elegans* which grow in warmer parts of east and south east of the country at altitudes up to 2,000 m (Ghahraman, 2004 ▶; Zargari, 1987 ▶). 

One of the most indicator species of *Boerhavia* is an Asian-African species that has usually been named *Boerhavia elegans *Choisy. The name *Boerhavia elegans *Choisy is attended by a reasonably well prepared and adequate description (Fosberg, 1978 ▶). In Iran,* B. elegans* found in Sistan & Baluchistan and Fars provinces (Ghahraman, 1996 ▶). External distribution of *B. elegans* is Djibouti; Ethiopia; Pakistan; Somalia, Kenya Northern Frontier Province (http://plants.jstor.org/flora/ftea003980).

"Sourkho" and "Sorhmard" are the vernacular names of *B. elegans* in Sistan & Baluchestan Province (Ramazani et al., 2010 ▶; Sadeghi et al., 2014 ▶). 

The anti-malaria effect of *B. elegans* was investigated by Ramazani et al., in 2010 ▶. No further biological properties have been reported in the literature for these species. From this viewpoint, the present study was carried out for the first time to evaluate the anti-oxidant activity of the plant and to determine the phenolic contents of different extracts of it.

**Botany**

**Figure 1:**
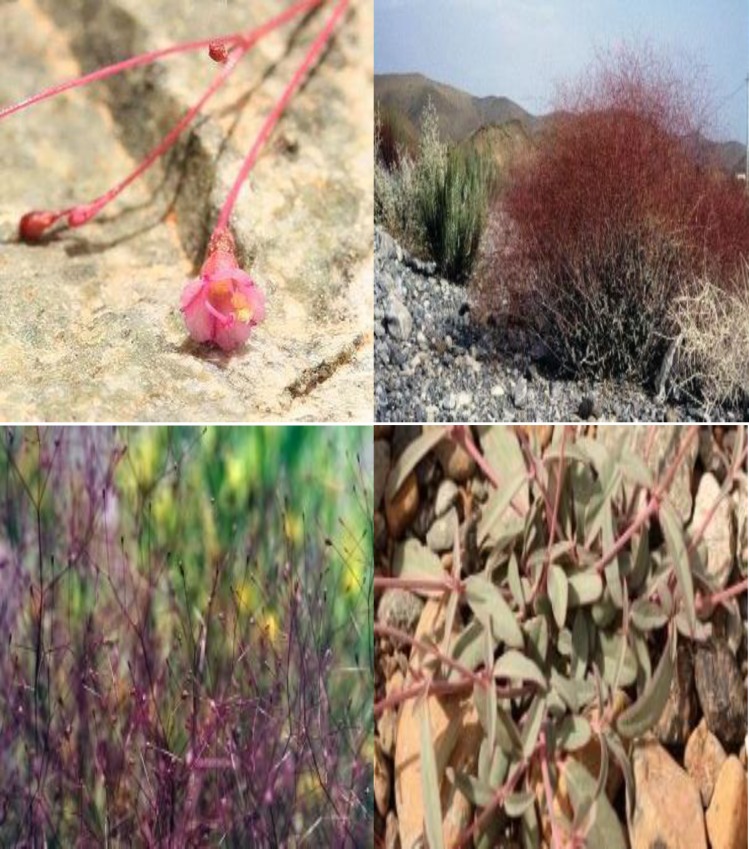
*B. elegans*; flower, shrub and leaf

*Boerhavia elegans* is a permanent plant with an erect glabrous shrub up to 1 m high and a burly root stock. Its fleshy stems become woody towards the base, which is dark green, often heated with red, glabrescent to pubescent, forking mainly from the base with the nodes ventricose. The leaves broadly are ovate-lanceolate or linear-lanceolate, green-gray above, pubescent-cansecent beneath, petiole 1cm long or absent; caulines sessile or shortly petiolate (Figure 1) (Chen et al., 2007 ▶; Ghahraman, 1996 ▶; Ramazani et al., 2010 ▶; Mahesh et al., 2012 ▶). 

Its flowers are solitary or geminate 2.5-3.5 mm long, pedicel long, cetaceous, and glabrous. Fruit characteristics of this herb is: oblong, clavi form, 5-angled, puberulent, grooved (Parsa, 1980 ▶). 

Also fruits of this species have a high content of mucilage when it is wet (Spellenberg, 2012 ▶). The flowering period of this species in Iran is March-April (http://plants.jstor.org/flora/ftea003980; Ghahraman, 1996 ▶).

**Table 1:** Scientific classificationof *B. elegans*

**Systematic classification**	***B. elegans***
Domain	Eukaryota
Kingdom	Plantae
Phyllum	Tracheophyta
Class	Spermatopsida
Subclass	Caryophyllidae
Order	Caryophyllales
Family	Nyctaginaceae
Genus	Boerhavia
Species	*elegans *- (Boiss.)
Subspecies:	*stenophylla*
Botanical name	*Boerhavia elegans stenophylla* (Boiss.)
Related Synonym(s)	*Boerhavia elegans* L. *stenophylla* (Boiss.) *Boerhavia* *Elegans Stenophylla* • *Boerhavia* *Rubicunda* *Stenophylla* Boerhavia elegans Choisy

**Taxonomic Description**

Table 1 shows taxonomic description and scientific classification of *B. elegans***.**

**Ethnobotanical information**

*B. elegans *has been used to treat dysmenorrhea, urinary tract diorders, intestinal infections, inflammation, jaundice, and body weakness in traditional medicine. Moreover, this species has expectorant, anti-diabetic, motive and diuretic properties and is used as the regenerator of heart and kidney (Ramazani et al., 2010 ▶). In Bamposht (East of Saravan), the Baluch tribes eat this plant as a vegetable and as an appetite stimulant. They use its decoction for removing fatigue and as an aphrodisiac (Sadeghi et al., 2013 ▶; Sadeghi et al., 2014 ▶). It is also used for inflammation in folk medicine (Zargari, 1987 ▶).

In the traditional medicine of Pakistan this species is called *Itsit*. Its leaves have a sharp taste and are appetizer, alexiteric; and used in opthalmia, eye wounds and pain of the joints. The fruits are expectorant, carminative, and useful in muscular pain, lumbago scabies and hasten delivery. The root is well known for its diuretic properties (Najam et al., 2008 ▶; Sandhu et al., 2011 ▶).

## Material and Methods

**Plant collection**

A voucher specimen (SCH-144) was deposited in the Higher Educational Complex of Saravan Herbarium.

**Preparation of extracts**

A portion of dried plant material (40 g) was extracted with methanol, chloroform and ethyl acetate using the soaking method for 48 h. After filtration and solvent evaporation, extracts were stored in sealed vials at 4 °C until biological testing. 

**Cytotoxic activity on two cell lines (PC3 and MCF-7)**

Primary cytotoxicity screening of* B. elegans* was done against 2 human cancer cell lines including PC3 (prostate cancer) and MCF7 (breast cancer). Cell lines were inseminated in a 96-well plate and incubated for 24–72 h. When cells reached >80% conﬂuency, they were incubated with crude extracts (Methanolic and dichloromethane, 50:50) at a concentration of 50 µg/ml dissolved in dimethyl sulfoxide (DMSO) at a maximum concentration of 0.05%. After 20, 44 and 68 h of incubation, 20 µl of Resazurin (Alamar blue) solution was added to each well and incubated at 37◦ C for 4 h, and then the absorbance was measured by ELISA reader at 600 nm. Doxorubicin was used as positive control, whereas cells incubated only with 0.05% of DMSO were used as a negative control and culture medium was used as background. The selectivity index (SI) of the extracts is deﬁned as the ratio of:

(SI = treated cells-untreated cells/background-untreated cells×100).

**Antioxidant evaluation**

*DPPH radical-scavenging activity*

The free radical scavenging activity of the plant extracts was performed according to the DPPH free radical method, described by Brand-Williams et al (1995) (Thaipong et al., 2006 ▶). 2.0 ml of different extracts with 500, 1000, 2000 μgr/ ml range of concentrations, was mixed with DPPH (0.1 mM) and 0.1 ml of extract solution (0.1 mg/ml) with methanol and, after 60 min standing; the absorbance of the mixture was measured at 517 nm against methanol as the blank on a UV/visible light spectrophotometer (UNICO UV 2100). Triplicate measurements were made and the radical scavenging activity was calculated by the percentage of DPPH that was scavenged using the following formula:

% Reduction = [(AB – AA)/AB] x 100

Where: AB: absorption of blank sample; AA: absorption of testing extract solution.

All analyses were run in triplicates and standard deviation (SD) was calculated.

**Ferric-Reducing antioxidant power (FRAP) assay**

The antioxidant capacity of plant extracts was done by iron reduction (FRAP assay) according to Benzie and Strain (1996) with some modifications. 300 mM acetate buffer pH 3.6, 10 mM TPTZ solution in 40 mM HCl, and 20 mM FeCl_3_ _ 6H_2_O solution were mixed for preparation of stock. FRAP reagent was prepared right away before analysis by mixing 25 ml acetate buffer, 2.5 ml TPTZ solution, and 2.5 ml FeCl_3_ _ 6H_2_O solution. Plant extracts (1000 μg/ml) were prepared by different solvent. 200 μg/ml of the extracts was mixed with 1.8 ml of the FRAP reagent and was incubated at 37 ºC for 30 min in the dark condition before using. Then readings of the colored products (ferrous tripyridyltriazine complex) were determined at 595 nm against distilled water blank. FeSO4-7H2O (100 - 1000 μM) was used for calibration. Ascorbic acid was used as positive control. Results are expressed mM Fe^2+^/ mg sample (Wojdyloet al., 2007 ▶; Chaouche et al., 2013 ▶).

**Determination of total phenolic contents (Matta and Giai, 1969)**

Total phenol content was determined by Folin-Ciocalteu reagent. A dilute solution of methanolic extract (0.05:1 g/ml) or Gallic acid (standard phenolic compound) was mixed with the Folin-Ciocalteu reagent (2.5 ml, 1:10 diluted with distilled water) and aqueous Na_2_CO_3_ (2 ml, 5%). The mixture was allowed to stand for 30 min and the phenolic contents were determined by colorimetry at 765 nm (Wojdylo et al., 2007 ▶). The total phenolic content was determined as mg of Gallic acid equivalent using an equation obtained from the standard Gallic acid calibration graph.

**Statistical analysis**

Anti-oxidant activities measured by DPPH and FRAP assays were done in triplicates to test the reproducibility of them. All results are presented as mean ± S.E. SPSS 15.0 (statistical software) was used for statistical analysis of results. Statistical analyses were performed by Student's t-test. The values of p <0.05 were considered statistically significant. Correlations among data obtained were calculated using Pearson’s correlation coefficient (r).

## Results

**Cytotoxic activity of** ***B. elegans***

In this work, primary cytotoxicity screening of* B. elegans* was done against 2 human cancer cell lines including PC3 (prostate cancer) and MCF7 (breast cancer). The results revealed that aerial parts of this plant (50µg/ml) inhibited 0% PC3 cells compared with Duxorubicin which inhibited 100% cell growth at concentration of 10µg/ml. 

Compared with Duxorubicin (10µg/ml, 95% inhibition),* B.elegans* (50µg/ml) showed 15.14% inhibition on MCF7 cell line. 

**Antioxidant activity**

In this study, cytotoxic and antioxidant activities as well as the total phenolic compounds were determined. Two methods of antioxidant assessment, DPPH and FRAP, were used for investigation of antioxidant activity of different extracts of *B. elegans*. Analyzing data obtained by these two methods could provide a more precise description of anti-oxidant activity.

**DPPH assay**

**Figure 2:**
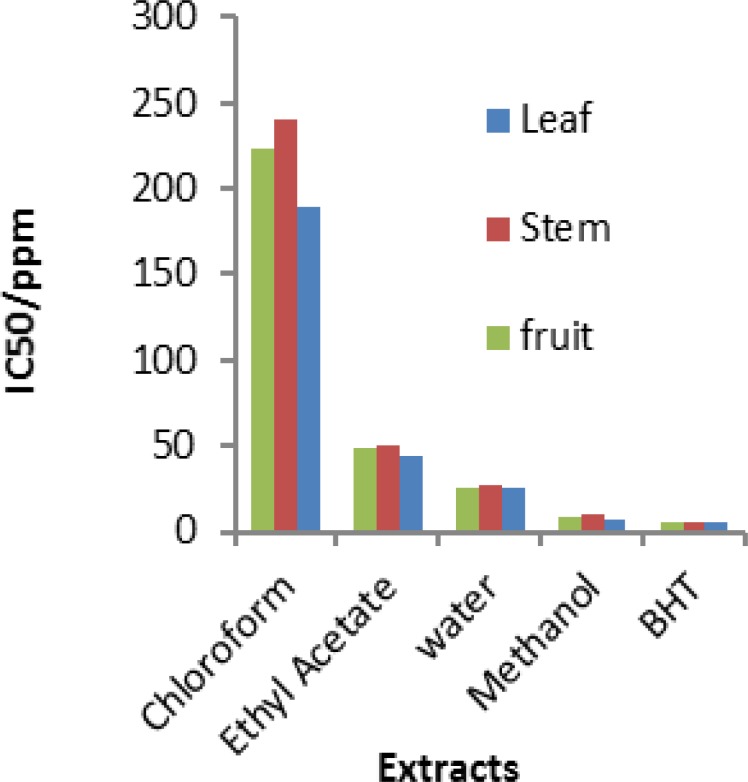
Concentration of different extracts required to reduce the initial DPPH radical by 50%

The results of DPPH radical-scavenging activity and FRAP assay of different extracts are shown in Figure 2. Considering the large variation of IC_50,_ values shows that IC_50_ ranged from 9.70 ppm in the methanolic extract to 223.21 ppm in chloroform extract. The methanolic extract of leaf had the highest antioxidant activity in the plant. This extract showed an exceptional anti-oxidant activity (IC_50_= 6.85 μg/ml) at 500 ppm, which was approximately near to BHT (IC_50_=4.8 μg/ml). While the lowest anti-oxidant activity is reported for Chloroform extract of the stem (IC_50_= 240.19 μg/ml) at 2000 ppm. 

**FRAP assay**

All analyzed extracts demonstrated significant antioxidant capacities with FRAP test. The methanolic extract of leaf showed 56.18. mMFe^2+^/mg sample values comparable to reducing power of ascorbic acid (60.75 mMFe^2+^/mg sample). Table 2 shows the antioxidant activity as IC_50_ (μg/ml) for DPPH assay or Fe (μg/ml) for FRAP assay of various types of *B. elegans* extracts.

**The phenolic content of the extracts**

In this study the concentration of total phenolic content (mg/g dry weight) of leaves, stems and fruits were measured (Table 3). The Phenolic content of plant materials, calculated as Gallic acid equivalent. The highest amount of phenolic content was found in leaves (16.41 mg/g) and decreased in the order of leaf > fruit > stem.

The percent deterrence of DPPH radical by the extracts was compared to a known synthetic antioxidant, Butylated Hydroxy-toluene (BHT) (Bhatt et al., 2012 ▶).

**Table 2:** Antioxidant activity as IC50 (μg/mL) for DPPH assay or FE (μg/mL) for FRAP assay of *B. elegans* extracts (mean±SD)

**Parts of the plant**	**Extract **	**DPPHIC50 (μg/ml)** **(mean±SD) **	**FRAP**
**leaf**	Water	24.76±0.98	36.63±0.28
	Methanol	6.85±0.62	56.18±79
	EthylAcetate	44.33±1.12	24.64±0.90
	Chloroform	189.33±0.21	15.33±0.65
**stem**	Water	27.12±0.83	30.58±0.91
	Methanol	9.70±0.17	47.79±0. 4
	EthylAcetate	50.79±1.40	24.64±1. 27
	Chloroform	240.19±0.20	23.65±0. 77
**fruit**	Water	26.18±0.33	33.50±0. 3
	Methanol	8.83±0.67	50.92±0. 49
	EthylAcetate	48.17±1.08	23.93±0. 34
	Chloroform	223.60±2.56	10.43±0. 45

**Table 3:** Total phenolic content expressed as mg Gallic Acid/g D.w (dry weight)

**Total phenolic contents**		
Leaf	Stem	Fruit
16.41± 0.89	12.48±0.54	14.31±0.58

## Discussion

Despite the wide therapeutic use of B. elegans, there are still lacks of scientific data in the literature which can clearly demonstrate the existence of its biological activities and explain the mechanism of its action. Ramazani et al, (2010) ▶ investigated anti-malaria effect of crude ethanolic extract of B. elegans that had promising anti-plasmodial activity. B. elegans was found to be highly active with IC50 values of < 20 μg/ml against P. falciparum strains (Ramazani et al., 2010 ▶). The ethanolic extract of *B. elegans* was screened against brine shrimp nauplii by Ramazani et al, (2010) ▶ as well as. The result showed that this species didn’t have significant toxicity (LC_50_> 1,000 μg/ml)*.* In this study compared with Duxorubicin (10µg/ml, 95% inhibition),* B.elegans* (50µg/ml) showed 15.14% inhibition on MCF7 cell line. These findings are inconsistent with the results obtained by Ramazani et al.

Medicinal plants that traditionally used in folk medicine are particularly interesting for investigation of their antioxidant effects. A number of methods and modifications have been proposed for anti-oxidant activity assessment. Total anti-oxidant activity, metal chelation, radical scavenging (DPPH) effects and reducing power as well as activities destructive to active oxygen species such as the superoxide anion radical, hydroxyl radical, and hydrogen peroxide are mainly used for this aim (Aksoy et al., 2013 ▶). In this study, the methods DPPH and FRAP were used. 

 The 1,1-Diphenyl-2-picrylhydrazyl (DPPH) radical is a stable organic radical with a maximum absorption at 517 nm. When alcoholic DPPH• solutions reacts with a hydrogen donating antioxidant, it is reduced to non-radical form of DPPH-H and color changes from deep-violet to light-yellow (Koleva et al., 2002 ▶). The antioxidants present in plants reduced colorless Fe^III^-TPTZ complex to Fe^II^-TPTZ, a blue colored compound in FRAP method (Katalinic et al 2004 ▶). Colour density shows the anti-oxidant potency of the extract. The anti-oxidant activity was correlated with the amount of total phenolic content present in the respective extracts in each assay. Methanolic and aqueous extract were proved to be the most efficient solvents for the extraction of anti-oxidants from *B.elegans* leaves, fruits, and stem.

Enzymes (such as superoxide dismutase, catalase, glutathione peroxidase, etc.), high (proteins) and low (polyphenols) molecular weight compounds, mineral elements (selenium, copper, chromium, Zink, etc.) and vitamins (like vitamin A, C and E) are well known compounds that play a crucial role in interception preoxidation damage in the biological system (Gupta et al., 2006 ▶). However, the chemical constituents present in the extract, which are responsible for anti-oxidant activity, need to be investigated. But it is obvious that the phenolic compounds in the extract may be responsible for such activities. Determination of total phenolic compounds showed that the observed antioxidant activity may be due to the presence of any of these constituents. 

Phenolic compounds are the most important groups of secondary metabolites in medicinal herbs and dietary plants that characterized by at least one aromatic ring (C6) bearing one or more hydroxyl groups. Various phenolic compounds (e.g., benzoic and cinnamic acid, coumarins, tannins, lignins, lignans, flavonoids and etc.) possess a diverse range of beneficial biological functions, including antioxidant activity. Unique structure and a high tendency of phenolic compounds for metal chelation and their redox properties allow them to act as reducing agents, hydrogen donators, and singlet oxygen quenchers which could lead to anti-oxidant activity (Khoddami et al., 2013 ▶; Bhatt et al., 2012 ▶; Huda-Faujan et al., 2009 ▶; Huang et al., 2009 ▶; Morgan et al., 1997 ▶). The findings of this study showed a positive relationship between high anti-oxidant activity and phenolic content for different parts of this plant. This is why phenolic compounds are known as hydrophilic antioxidants. According to exceptional high anti-oxidant activity of leaves with high phenolic content, further research on phenolic compounds in this species seems necessary. Although nonphenolic compounds such as trace elements may also decrease the antioxidant activity (Souri et al 2008 ▶). It was also found a high correlation (r=1.00, P<0.01) between anti-oxidant activities as determined by DPPH assay for various parts. There was no correlation between antioxidant activity as determined by DPPH and FRAP assays (r= -0.60 to -0.85, P>0.05). This might be due to the potential of an antioxidant against free radicals and inevitably doesn’t equal with its ability to reduce ferric to ferrous. Since there are some problems with the FRAP assay regarding to color interference and variable rates of reaction point, the DPPH assay is the preferred assay in preliminary screening of extracts of plants. The DPPH method is very rapid, simple, sensitive, reproducible and does not require special instrumentation (Koleva et al., 2002 ▶; Clarke et al., 2013 ▶). 

In the present study, methanolic extract of *B. elegans* bears comparable antioxidant activity to the standard compounds. The aqueous extract also showed good antioxidant activity. Its constituents scavenge free radicals, chelate the catalytic metal ions, and may exert a protective effect against oxidative damage induced to cellular macromolecules. Free radicals are often generated as byproducts of biological reactions or from exogenous factors. The involvements of free radicals in the pathogenesis of a large number of diseases are well documented. A potent scavenger of free radicals may serve as a possible preventative intervention for the diseases (Ebrahimzadeh et al., 2008 ▶). 

*B. elegans* contains high volume of phenolic compound which may be responsible for the antioxidant properties. This plant exhibited strong anti-malaria effect. 

These properties may be due to its antioxidant activity. The potent antioxidant activity of *B. elegans* supports its possible use as a natural antioxidant in food industries and other pharmaceutical preparations. In summary, the present study showed that the biologically active constituents from *B. elegans* can be extracted with different solvents. Further experiments are necessary to identify the metabolites responsible for pharmacological activities of the crude methanolic extract.
